# Development of improved therapeutic mesothelin-based vaccines for pancreatic cancer

**DOI:** 10.1371/journal.pone.0193131

**Published:** 2018-02-23

**Authors:** Michael White, Andrew Freistaedter, Gwendolyn J. B. Jones, Emmanuel Zervos, Rachel L. Roper

**Affiliations:** 1 Department of Surgery, Brody School of Medicine, East Carolina University, Greenville, NC, United States of America; 2 Department of Microbiology, Brody School of Medicine, East Carolina University, Greenville, NC, United States of America; University of South Alabama Mitchell Cancer Institute, UNITED STATES

## Abstract

Pancreatic cancer is the 5^th^ leading cause of cancer deaths, and there are no effective treatments. We developed a poxvirus platform vaccine with improved immunogenicity and inserted the mesothelin gene to create an anti-mesothelin cancer vaccine. Mesothelin expression is mostly restricted to tumors in adult mammals and thus may be a good target for cancer treatment. We show here that the modified vaccinia virus Ankara (MVA) virus expressing mesothelin and the enhanced MVA virus missing the immunosuppressive A35 gene and expressing mesothelin were both safe in mice and were able to induce IFN-gamma secreting T cells in response to mesothelin expressing tumor cells. In addition, the MVA virus has oncolytic properties in vitro as it can replicate in and kill Panc02 pancreatic adenocarcinoma cell line tumor cells, even though it is unable to replicate in most mammalian cells. Deletion of the A35 gene in MVA improved T cell responses as expected. However, we were unable to demonstrate inhibition of Panc02 tumor growth in immunocompetent mice with pre-vaccination of mice, boosts, or even intratumoral injections of the recombinant viruses. Vaccine efficacy may be limited by shedding of mesothelin from tumor cells thus creating a protective screen from the immune system.

## Introduction

Pancreatic cancer is the 5^th^ leading cause of cancer deaths in the United States [1] and has the lowest 5-year survival rate (7%) for solid tumors [2] largely owing to the fact that radiation, surgery, and current chemotherapy options are ineffective. New treatment strategies are essential. Human mesothelin is normally expressed only in mesothelial cells of the pleura, peritoneum, and pericardium. However, mesothelin is overexpressed in a high percentage of ovarian cancers, pancreatic cancers, non–small lung cancers, and mesotheliomas, making it a potential target for anti-cancer treatments [3–7]. Human and mouse mesothelin share sequence similarity, expression patterns, and biochemical characteristics, [7], and the homeostatic function of mesothelin in mammals is unknown: the gene can be deleted without apparent effect in mice [8]. Human mesothelin has been proposed to be a malignancy factor as it increased tumor cell proliferation and migration *in vitro* and tumor size in nude mice [5, 7], and siRNA specific for mesothelin suppressed tumor growth in a rat renal carcinoma model [9]. However, we have shown that stable overexpression of mesothelin in a pancreatic cancer cell line did not increase cell proliferation or anchorage- independent growth *in vitro*, suggesting that mesothelin is not necessarily a tumor progression factor [7]. Indeed, we tested the effect of mesothelin expression in the presence of an intact host immune system and found that overexpression of mesothelin unexpectedly decreased metastases and even inhibited tumor formation *in vivo* in immunocompetent mice [7]. This suggested that an immune response to mesothelin might be effective in reducing tumor growth.

Tumor associated antigens are of interest as targets because they are expressed on cancerous cells, but not on most normal adult tissues. A number of studies, including Phase 1 and II clinical trials, are ongoing to target mesothelin expressing cancers [5, 10–14]. Chimeric antibodies, immunotoxins, and drug conjugates that bind mesothelin are being tested as well as chimeric antigen receptor T-cells (CAR T cells) that bind and kill mesothelin-expressing cells. Live attenuated *Listeria monocytogenes*-expressing mesothelin is being evaluated as an anti-cancer vaccine [5, 10–14]. Both antibodies and T lymphocytes have been shown to have protective anti-mesothelin activity [5, 10, 13–15]. This fact may be especially useful because irradiation of tumor cells enhances the expression of mesothelin [14], so that an anti-mesothelin vaccine may be an effective combination with irradiation. The most well-characterized recombinant immunotoxins against mesothelin are SS1P and monoclonal antibodies are MORAb-009 [16]. A phase I study of SS1P given as a bolus i.v. infusion to 2 patients with mesothelin-expressing pancreatic cancers was published in 2007 showing safety and feasibility but, unfortunately, no clinical response [17]. Additional high affinity monoclonal antibodies have been developed which bind to alternative regions of the mesothelin protein, allowing for additional mesothelin targeting in the presence of MORAb-009/SS1 or MUC16 [18].

Several poxviruses are used as recombinant vectors to develop vaccines for a wide range of infectious diseases including bacterial, viral and parasitic pathogens, as well as for cancer therapies, and several are approved or currently in clinical trials [19–26]. The only HIV vaccine with demonstrated efficacy in humans to date employs an avian poxvirus vector, and it entered NIH-sponsored Phase 2b/3 trials in 2016 [27]. Poxviruses such as vaccinia virus are suitable as vectors because they are easily grown to high titer in a wide variety of animals and cell types, can accommodate insertions of large pieces of DNA into their genomes, are very stable even when dried, and induce robust B and T cell immunity [19, 28–30]. Vaccinia virus is the most commonly used poxvirus vaccine vector; however, its use is limited by its potential virulence, especially in immunocompromised hosts [19, 21, 29, 30]. Raccoonpox virus provides a safer vector compared to vaccinia virus [19, 21, 26, 30], and the Modified Vaccinia virus Ankara (MVA) strain is even more attenuated, because it cannot replicate in most mammalian cells [31]. However MVA immunogenicity has been limiting, requiring high doses and boost vaccinations [28, 31–38]. Poxviruses have been shown to have efficacy as a vaccine platform against cancers [19, 39, 40], but patient responses still need to be improved. In order to strengthen immune responses and treatment outcomes, we have developed a recombinant MVA (MVAA35Del) that is missing the immunosuppressive A35 virulence gene that blocks antigen presentation and B and T lymphocyte responses [29, 31, 41–43]. Removal of the A35 gene improves antibody and cytokine production, numbers of responding T lymphocytes, and immunoglobulin class switching [29, 31].

In the current study, we assess the safety, immunogenicity, and protective efficacy of a therapeutic MVA virus vaccine expressing mesothelin (MVAmeso) in a heterotopic pancreatic tumor model using the pancreatic adenocarcinoma cell line Panc02 that expresses mesothelin in culture and in tumors in the mouse model [7]. The Panc02 tumor line was derived from C57Bl6 mice and thus can be grown in syngeneic mice to allow for study of an anti-tumor immune response in an immunocompetent mouse model. This has a great advantage over tumor studies performed with xeno- or allografts in immunocompromised mice. To determine whether we can improve vaccine immunogenicity and efficacy, we test the parental normal MVAmeso virus vaccine against the MVA mesothelin expressing virus that has had the immunosuppressive A35 gene removed (MVAmesoA35Del).

## Materials and methods

All animal experiments were conducted with the approval of the East Carolina University Animal Care and Use Committee (K157) in an AAALAC accredited animal facility. Isoflurane anesthesia was used for tumor injections and for euthanasia.

### Cell lines and virus

The C57BL/6 chemically-induced pancreatic adenocarcinoma cell line, Panc02, was a kind gift from Dr. Keping Xie (MD Anderson Cancer Center, Houston, TX). Panc02 cells were grown in RPMI, with 10% Fetal Bovine Serum, 2 mM glutamine, 100 U/ml of penicillin and streptomycin. Mesothelin over expressing Panc02 cell lines are described in Zervos, et al [7]. Cells were incubated at 37°C with 5% CO_2._ Vaccinia virus, MVA, and the MVA A35 deletion mutant viruses were previously described [29, 31]. Baby hamster kidney (BHK) cells were employed for growth of MVA and were cultured using Dulbecco's Modified Eagle Medium (DMEM), supplemented with 10% Fetal Bovine Serum, 2 mM glutamine, 100 U/ml of penicillin and streptomycin.

### Mesothelin MVA virus construction

In order to create a therapeutic anti-mesothelin vaccine, we inserted mouse mesothelin into the MVA viral genome. We used the normal wild type MVA or the MVA in which the A35 virulence gene had been deleted [31] to create the MVAmeso virus and the MVAmesoA35Del recombinant viruses respectively. In both cases, mesothelin was cloned into a vector (pLW44, a gift from L. Wyatt, National Institutes of Health) designed for insertion into an area of the MVA genome where genes had been naturally deleted during passage of the virus [44]. Recombinant viruses expressing the gfp marker protein were selected, purified, and analyzed to confirm the genotype by raid prep PCR analysis [45]. The presence of the mesothelin gene was confirmed using mesothelin-specific primers (GGCTGGCTATGGCTGTAAGAC and AGCAGTGTTGGATGAGTCCATCGT) with an annealing temperature of 51° C. In order to assess the presence of the A35 gene, recombinant viruses were analyzed using A35 specific primers (TGACTATAACAGATACATT and CAGATTATCTGAAGGATTGAT) at an annealing temperature of 40° C. To confirm the insertion sites of the mesothelin, we used primers in the MVA flanking regions, (CATAAGTATAAAGTCCGACTATTGTTC and GATAAAGTTGCATCATCACCTAT). The PCR amplicons were run on an 0.8% agarose gel.

### Rabbit anti mouse mesothelin anti-peptide antibody

Peptides representing murine mesothelin coding sequence, GVYGFQVSEADVRALGGLAC and CPPGKEPYKVDEDLIFYQN, were synthesized and conjugated to KLH carrier proteins via the terminal cysteine residues (underlined, Genemed Synthesis), and used for immunization of two rabbits [7]. Sera were confirmed for reactivity with the immunizing peptides by ELISA.

### Western blot

BHK cells (2x10^6^cells/well) were uninfected or infected with the 3 MVA viruses at a multiplicity of infection (MOI) of 5 plaque forming units (pfu, infectious virus particles) of virus per cell for 13 h in 5% CO2 atmosphere. Cells were lysed with disruption buffer and heat inactivated at 95^°^C for five minutes. DNA was sheered using a 22-gauge needle and samples were loaded onto a pre-cast 4–20% gradient gel (Thermo Scientific) for SDS-PAGE and transferred overnight. Membrane was blocked for 30 minutes at room temperature with 1% fat free milk in TBS, and incubated with rabbit anti-mesothelin antibody (1:10,000) for 2 h at room temperature, washed, and then incubated in anti-rabbit IgG (Fc) AP Conjugate (Promega), 1:7500 at room temperature for one h, washed and developed with Western Blue stabilized substrate (Promega). HEK transected cells, previously described [7], were used as control. For analysis of released mesothelin, supernatants were collected from wells containing the normal or mesothelin over-expressing Panc02 cells [7], uninfected BHK cells or cells infected with MVA viruses. The cell supernatants were run on an SDS-PAGE gel, transferred to a PVDF membrane and probed with a rabbit anti-mesothelin antibody and an alkaline phosphatase conjugated 2° antibody as described above.

### Infection of mice with MVAmeso virus

Female C57BL/6 mice (Charles River, 10–14 weeks of age) were used for experiments. All mice were housed in the East Carolina University Department of Comparative Medicine AAALAC accredited animal facility and kept in conventional conditions with full access to food and water throughout the study. All procedures were approved by the Institutional Animal Care and Use Committee and in accordance with recommendations for proper care and use of laboratory animals. In order to assess the safety of injecting MVA virus expressing mesothelin, we injected mice i.m. (n = 5) with PBS, normal wild type MVA, MVAmeso, or MVAmesoA35Del (3.8 x 10^7^ pfu/mouse i.m. in 25 ul, and boosted 1 month later with 1.5 x 10^7^ pfu/mouse) and monitored mouse health daily by body score condition and weight up to 125 days, similar to previous work [29].

### IFN-gamma mesothelin ELISPOT

Numbers of gamma interferon (IFN-gamma)-secreting spleen cells were enumerated similarly to methods described previously [29, 31]. Ninety-six-well plates (Immulon H2B; Thermo Electron) were coated overnight with purified rat anti-mouse IFN-gamma (1 mg/ml; Pharmingen) at 4°C. Plates were washed with blocking buffer before adding a titration of murine splenocytes in RPMI 1640 medium. Splenocytes were harvested from PBS, MVA, MVAmeso, or MVAmesoA35Del infected mice 1 month after vaccination. The *in vitro* stimulation of splenocytes was achieved either by the addition of Panc02 cells, wild type vaccinia virus (MOI = 3), or Lewis Lung cells, which do not express mouse mesothelin [7], followed by incubation for 40 h at 37°C. Plates were then washed (PBS with 0.05% Tween 20, 0.1% Sodium Azide) and incubated with biotinylated rat anti-mouse IFN-gamma (0.5 mg/ml; Pharmingen) for 2 h at 37°C. Plates were washed and incubated with streptavidin-AP for 1 h at 37°C. Plates were developed with an agarose-BCIP (5-bromo-4-chloro-3- indolylphosphate)-AMP mixture, and spots were counted by using a dissection microscope.

### MVA replication

To assess viral replication over time [41, 42], 350,000 cells were plated in a 24-well plate. BHK cells were used as a positive control since MVA is known to replicate in them. BHK or Panc02 cells were infected with MVA at an MOI of 5 pfu/cell for 2 h, then inocula were removed and the cells were washed once. Supernatants and cells were harvested separately at six time points: 0, 12, 16, 24, 36, and 48 h post-infection. These samples were frozen and thawed three times before being titered in BHK cells in 6-well plates [31].

### MVA killing of Panc02cells

Panc02 cells (10,000 cells/well) were plated in triplicate in a 96-well plate. MVA was added to the wells at a multiplicity of infection (MOI) of 0.5 and 5.0 pfu/cell. After 24 h, 10 ul 3-(4,5-dimethylthiazol-2-yl)-5-(3carboxymethoxyphenyl)-2-(4-sulphophenyl)-2H-tetrazolium, inner salt/phenazine methosulphate (MTS/PMS) (2.0 mg/ml MTS, Promega and 0.1 mg/ml PMS, Sigma) was added to each well, and the absorbance was read at 492 nm [42]. Media only was used to define the background control level.

### Tumor model

We used the syngeneic heterotopic tumor model employing the murine pancreatic adenocarcinoma Panc02 cell line [7]. Female C57BL/6 mice (Charles River, 10–14 weeks of age, n = 4–8 per group) were injected s.c. in the right flank with Panc02 cells (6.4x10^5^), one injection per mouse, under isoflurane (3%) inhalation anesthesia [7]. Tumor size was measured three times per week by a digital caliper, and volume was calculated as [(smallest diameter^2^) x largest diameter]/2. Mice were humanely euthanized if they reach endpoints of body condition score, tumor size, ulceration, or impairment. For analysis of safety and immunogenicity, mice were injected with 3.8 x 10^7^ pfu/mouse i.m. in 25 ul, and boosted 1 month later with 1.5 x 10^7^ pfu/mouse. For treatment of tumor bearing mice, mice were injected s.c. in the right flank with Panc02 cells (6.4x10^5^), and then injected i.m. contralaterally with 7.6 x 10^7^ pfu/mouse 25 ul 3 times, on days 11, 18, 25 after tumor cell injections. For pre-vaccination of mice, mice were injected i.m. with 3.8 x 10^7^ pfu/mouse, and then boosted with 1.5 x 10^7^ pfu/mouse, and challenged with Panc02 tumor cells as above. For viral injections into tumors, tumors were allowed to grow, and viral injections (3.5 x 10^6^ pfu/mouse in 25 ul) into tumors began when tumors were 100 mm^3^.

### Statistical analysis

Experiments were repeated at least three times, and representative data are shown. A two-tailed Student’s *t* test was used to compare groups. *P* values < 0.05 were considered significant.

## Results

### MVA-mesothelin expressing vaccine viruses

While tumor associated antigens, such as mesothelin, often do not induce a robust immune response, evidence suggests it is possible to generate an anti-tumor response by presenting the tumor antigen in an immunogenic context, such as by expressing the protein in a viral infection [19, 46]. In order to create a putative therapeutic anti-mesothelin vaccine, we inserted the mouse mesothelin gene into the poxvirus MVA genome under a viral promoter so that mesothelin would be expressed in any cells infected with the recombinant virus. We constructed both a normal wild type MVA virus expressing mesothelin (MVAmeso) and a mesothelin-expressing MVA virus in which the viral A35 immunosuppressive virulence gene had been deleted (MVAmesoA35Del) [31]. The purified viruses were analyzed by PCR to confirm the presence of the mesothelin gene in the expected loci in the genomes and the presence or absence of the A35 gene.

**Fig 1:**
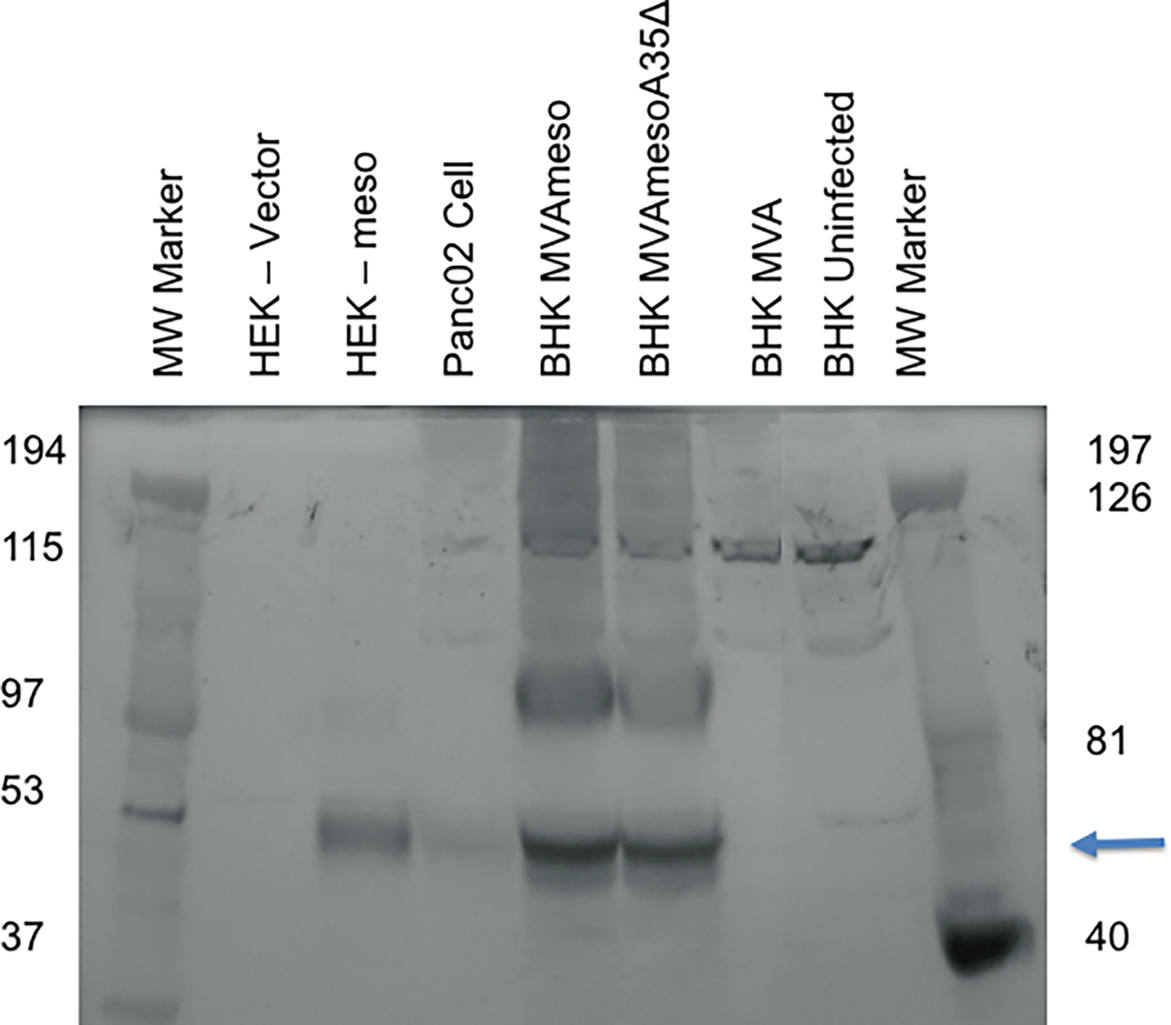
Mesothelin is expressed from MVAmeso viruses. BHK cells (2x10^6^ cells/well) were uninfected or infected with MVA, MVAmeso or MVAmesoA35Del. Cells were lysed and samples were loaded onto a pre-cast 4–20% gradient gel. Rabbit anti-mesothelin antibody and anti-rabbit IgG (Fc) AP Conjugate (Promega) detected mesothelin protein (arrow). HEK transected cells, previously described [7], were used as control.

Next, we wanted to confirm the expression of mesothelin protein from the recombinant viral genomes in infected cells. As shown in Fig 1, mesothelin protein was detected as a broad band ~50 kDa. Mesothelin is glycosylated, so differences in glycosylation can be seen as different MW bands, and glycosylated forms may vary in different cell types [7]. Mesothelin protein was strongly expressed in both MVA mesothelin virus-infected BHK cells, with monomers and apparent dimers (~100 kDa), but not in cells infected with MVA parental wild type virus or uninfected BHK cells (Fig 1). This indicated that the recombinant mesothelin expressing viruses were able to express significant quantities of mesothelin protein in infected cells.

As a control, mesothelin was detected by Western blot in HEK cells transfected with a mesothelin expressing plasmid with a strong promoter [7], but not in HEK cells transfected with the control plasmid vector. Mesothelin could also be detected in the Panc02 murine pancreatic adenocarcinoma cell line as we have shown previously by Western blot and flow cytometry [7]. While mesothelin is a tumor associated antigen that can be detected in Panc02 cells grown *in vitro* or as a tumor grown in the mouse, it is not a highly abundant protein [7].

**Fig 2:**
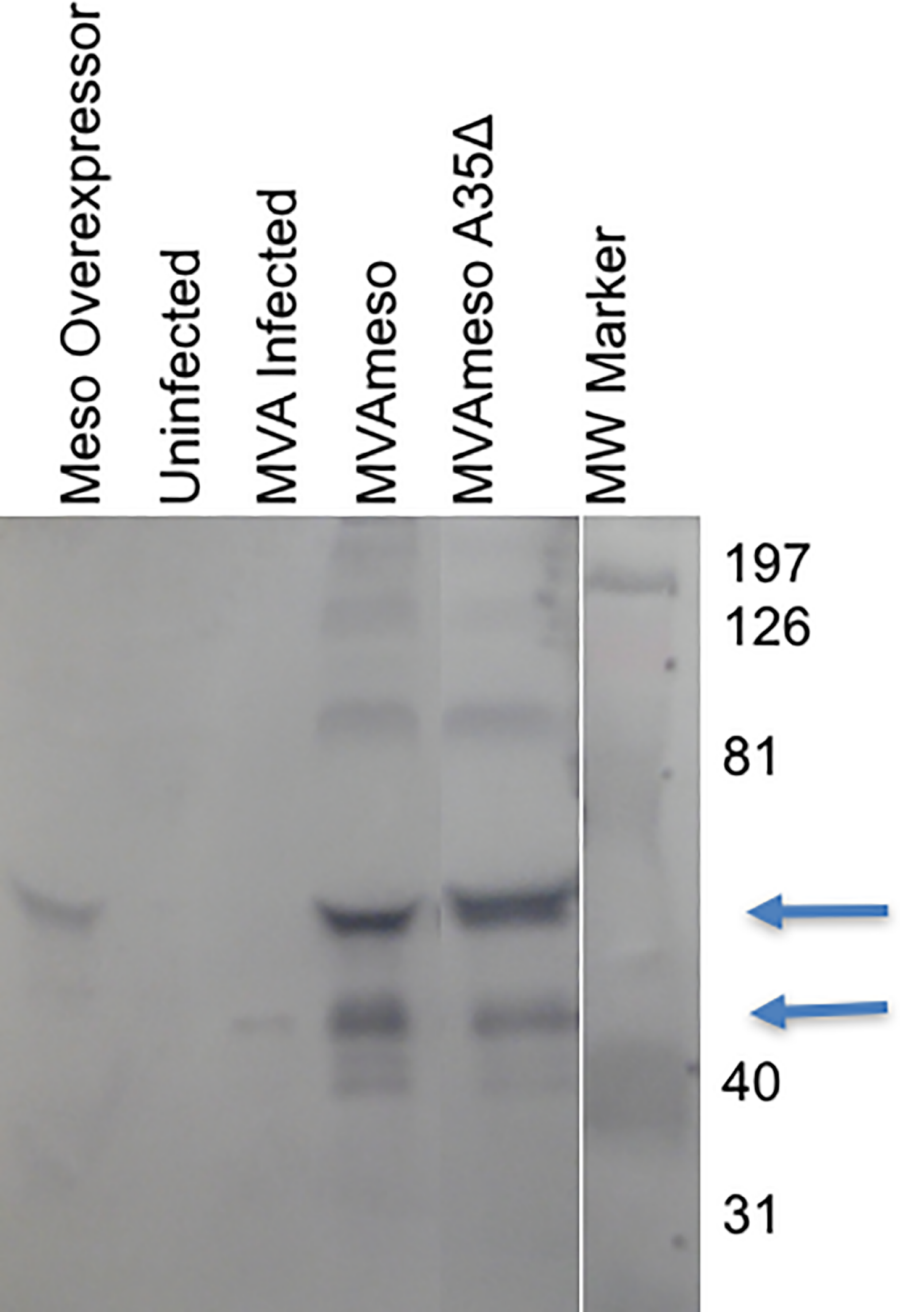
Mesothelin protein is released into supernatants. BHK cells (2x10^6^ cells/well) were uninfected or infected with MVA, MVAmeso or MVAmesoA35Del viruses. Supernatants were collected from wells containing the mesothelin over-expressing Panc02 cells “labeled Meso Overexpressor” [7], uninfected BHK cells, or BHK cells infected with MVA viruses and analyzed by Western blot using a rabbit anti-mesothelin antibody and anti-rabbit IgG (Fc) AP Conjugate (Promega).

Multiple forms and sizes of cleaved mesothelin proteins have been reported to be released from cells expressing it [6, 7]. We have previously shown that 2 forms of the mesothelin protein are found in supernatants of Panc02 cells, a carboxy terminal region released by furin cleavage and one derived from the amino terminus [7]. We assessed what forms of mesothelin proteins are present in the supernatants of BHK cells infected with the 3 MVA viruses (wild type MVA, MVAmeso, and MVAmesoA35Del) and for comparison cultured Panc02 cells overexpressing mesothelin [7]. As seen in Fig 2, several protein bands that react with rabbit anti mesothelin antisera were detected by Western blot, with large quantities of released mesothelin apparent in the cells infected with the recombinant viruses encoding mesothelin.

### Recombinant MVA mesothelin safety

**Fig 3:**
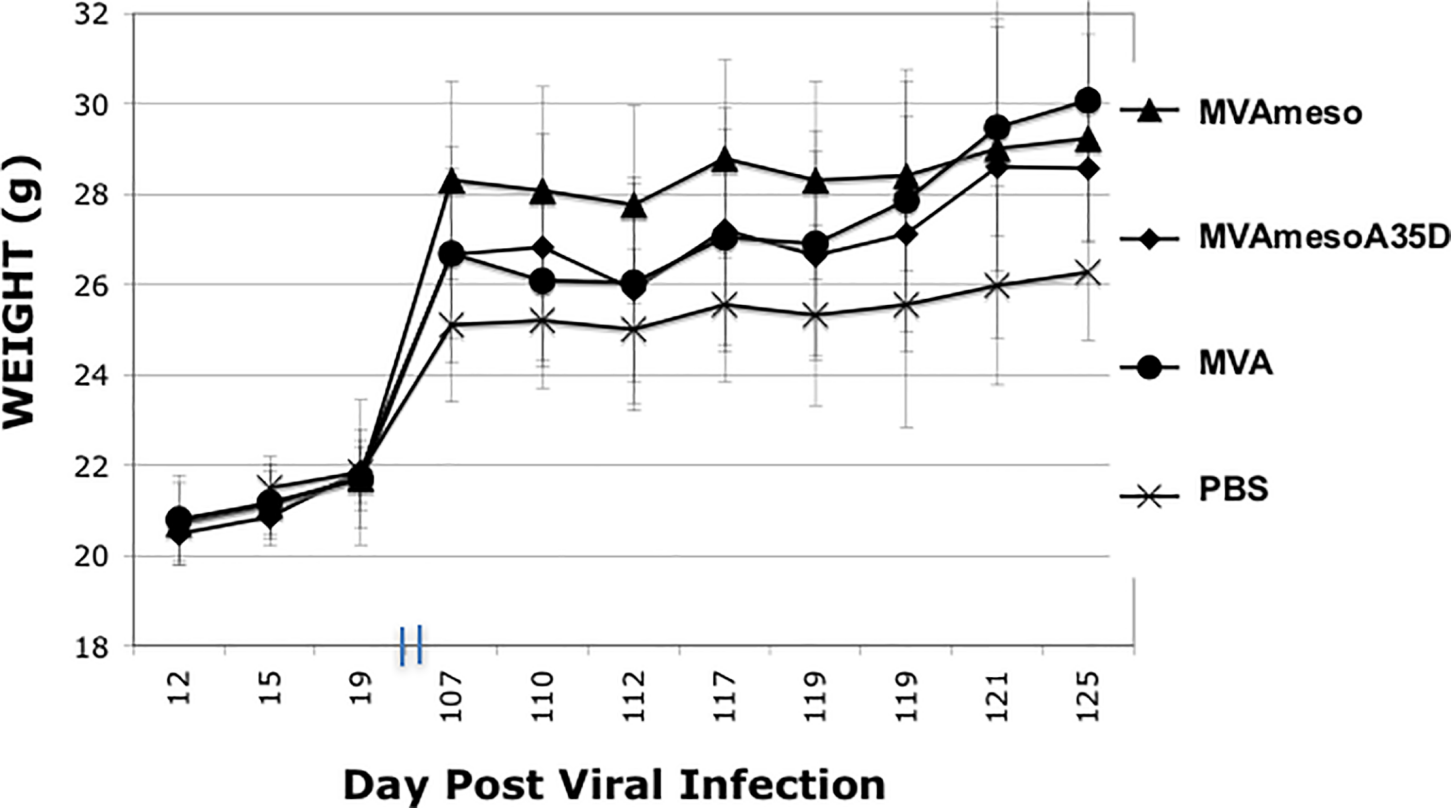
MVA meso virus injections are safe. Female C57BL/6 mice (n = 5) were injected i.m. with PBS, MVA, MVAmeso or MVAmesoA35Del and monitored daily by body score condition (no change) and weight up to 125 days. No adverse effects were noted. Average weights are shown + SEM.

In order to assess the safety of injecting MVA viruses expressing mesothelin, we injected mice i.m. (n = 5) with PBS, wild type MVA, MVAmeso, and MVAmesoA35Del. Mice were boosted 1 month later. We monitored mouse health by body score condition daily and weight for 125 days as shown in Fig 3. There were no significant differences between the 4 groups. We observed no adverse effects after the vaccination. This suggests that no significant anti-mesothelin autoimmune disease was induced in the mice.

### Immune response

**Fig 4:**
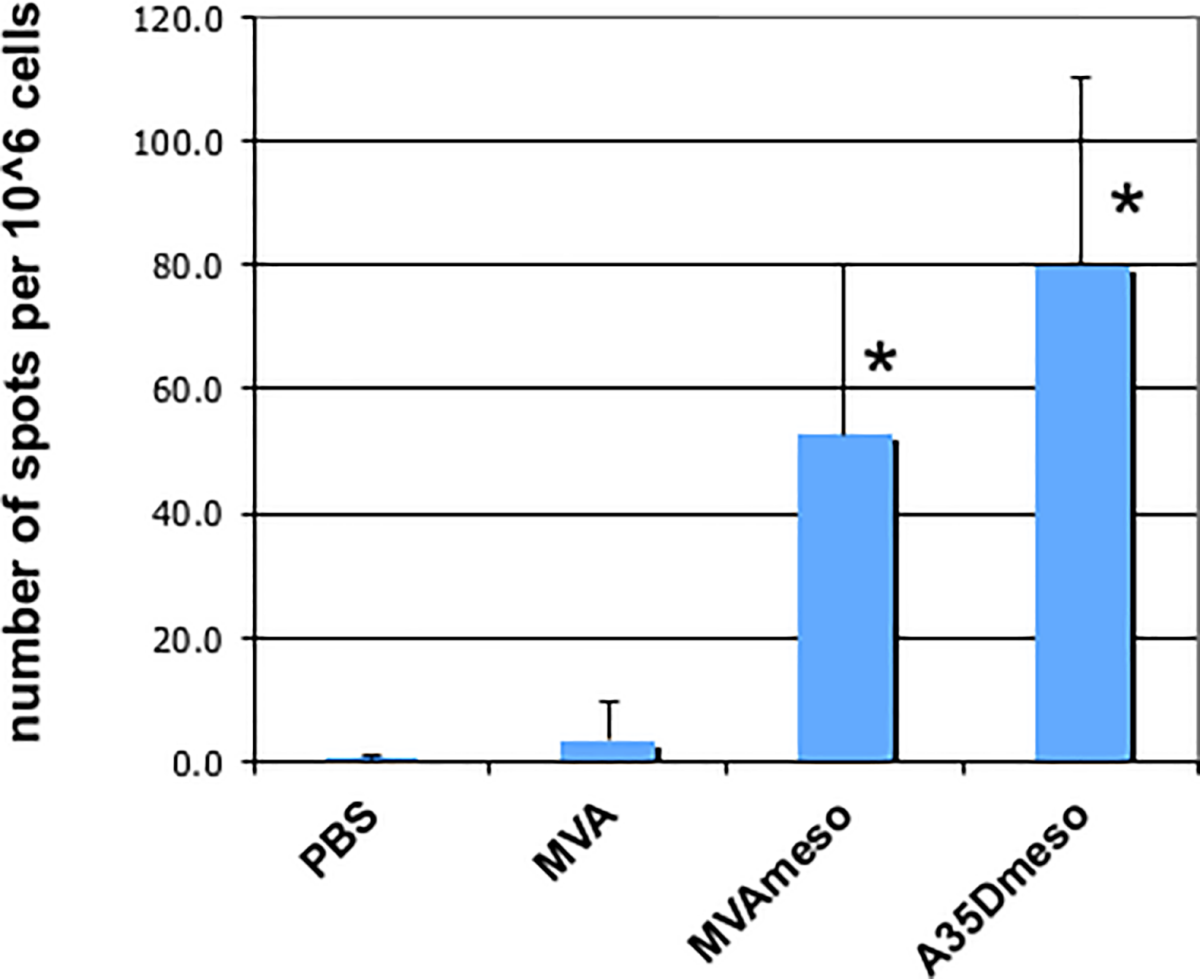
Mesothelin expressing viruses induce IFN-γ responses to Panc02 cells. Mice (n = 5) were infected with MVA, MVAmeso, MVAmesoA35Del (A35Dmeso), or mock infected with PBS, and splenocytes were harvest 1 month later. 100 live Panc02 cells were added to splenocytes and incubated with Panc02 cells for 40 h at 37°C. Plates were then washed and incubated with biotinylated rat anti-mouse IFN-gamma and streptavidin-AP and developed with BCIP. Average numbers are shown + SEM. * MVAmeso and MVAmesoA35Del vaccinated mice had significantly more responding splenocytes (p<0.05) than MVA and PBS treated mice.

To determine whether the viruses expressing mesothelin protein were able to induce an immune response in mice, we first attempted to measure anti-mesothelin antibody in vaccinated mouse sera. We attempted to measure reactivity to Panc02 cells, which express mesothelin on the surface by flow cytometry as we have done previously [7], however we got very low responses, possibly due to released mesothelin (Fig 2) binding the anti mesothelin antibodies *in vivo*. We then measured the frequency of anti-mesothelin IFN-gamma secreting T lymphocytes by ELISpot assay. Mice vaccinated with PBS or MVA had very few (<5) splenocytes that secreted IFN-gamma in response to Panc02 cells (Fig 4). MVAmeso vaccinated mice had an average of 53 IFN-gamma secreting T cells per 10^6^ splenocytes, and MVAmesoA35Del had an average of 80, significantly more (p<0.05) than MVA and PBS treated mice. In comparison, there were very few spots (1–3) in response to stimulation with Lewis Lung cells that do not express mouse mesothelin, and mice vaccinated with MVA, MVAmeso and MVAmesoA35Del viruses all had good responses to restimulation with vaccinia virus (124, 147, and 148 spots respectively). These results indicate that while all mice responded to the virus infection, and the mesothelin expressing viruses induced an immune response in mice that reactivated in response to mesothelin expressing Panc02 cells. Additionally, vaccination with the MVAmesoA35Del virus induced 52% more responding T lymphocytes compared to the MVAmeso virus, suggesting that removal of the A35 induced a stronger immune response, but the difference did not reach statistical significance.

### MVA replication in Panc02 cells

**Fig 5:**
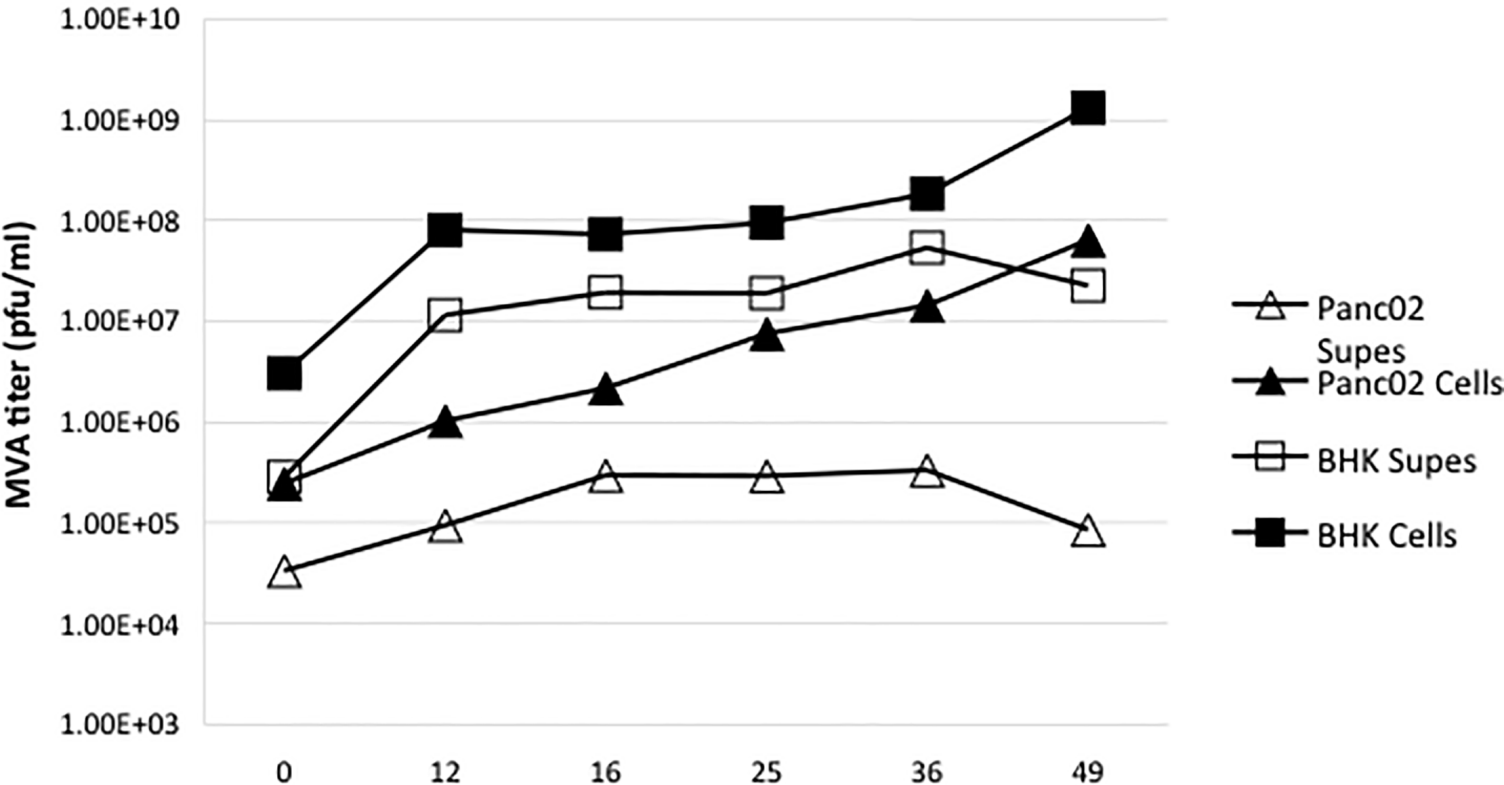
MVA replicates in Panc02 cells. 350,000 BHK or Panc02 cells were plated in a 24-well plate and infected with MVA at an MOI of 5. Supernatants and cells were harvested separately at six time points: 0, 12, 16, 24, 36, and 48 h post-infection. These samples were frozen and thawed three times before being titered in BHK cells in 6-well plates.

Some oncolytic poxviruses have been shown to specifically target and replicate in tumor tissues *in vivo* [19] because the tumor or microenvironment supports viral replication. We therefore wanted to assess if MVA virus was able to infect and replicate in Panc02 cells by a one-step viral growth curve [47]. MVA is unable to replicate in almost all mammalian cells: baby hamster kidney (BHK) cells are one known exception in which MVA can replicate. BHK and Panc02 cells were infected at an MOI of 5 pfu/cell so that each cell would be infected, and the supernatants and cells were harvested separately and titered for virus quantity over time. As shown in Fig 5, over 48 h the number of infectious MVA virions (plaque forming units, pfu) increased in BHK cells more than 2 logs. MVA replication in Panc02 cells also increased by more than 2 logs, suggesting that the MVA viruses might be effective oncolytic viruses *in vivo* since they can infect and replicate selectively in the Panc02 tumor cells, while unable to replicate in normal mammalian tissues. Viruses released into the supernatants also increased during MVA infection of both cell lines. These results were promising, because they indicated that the virus could potentially amplify *in situ* and spread to cells that were not initially infected at the time of virus inoculation.

### MVA can kill Panc02 cells

**Fig 6:**
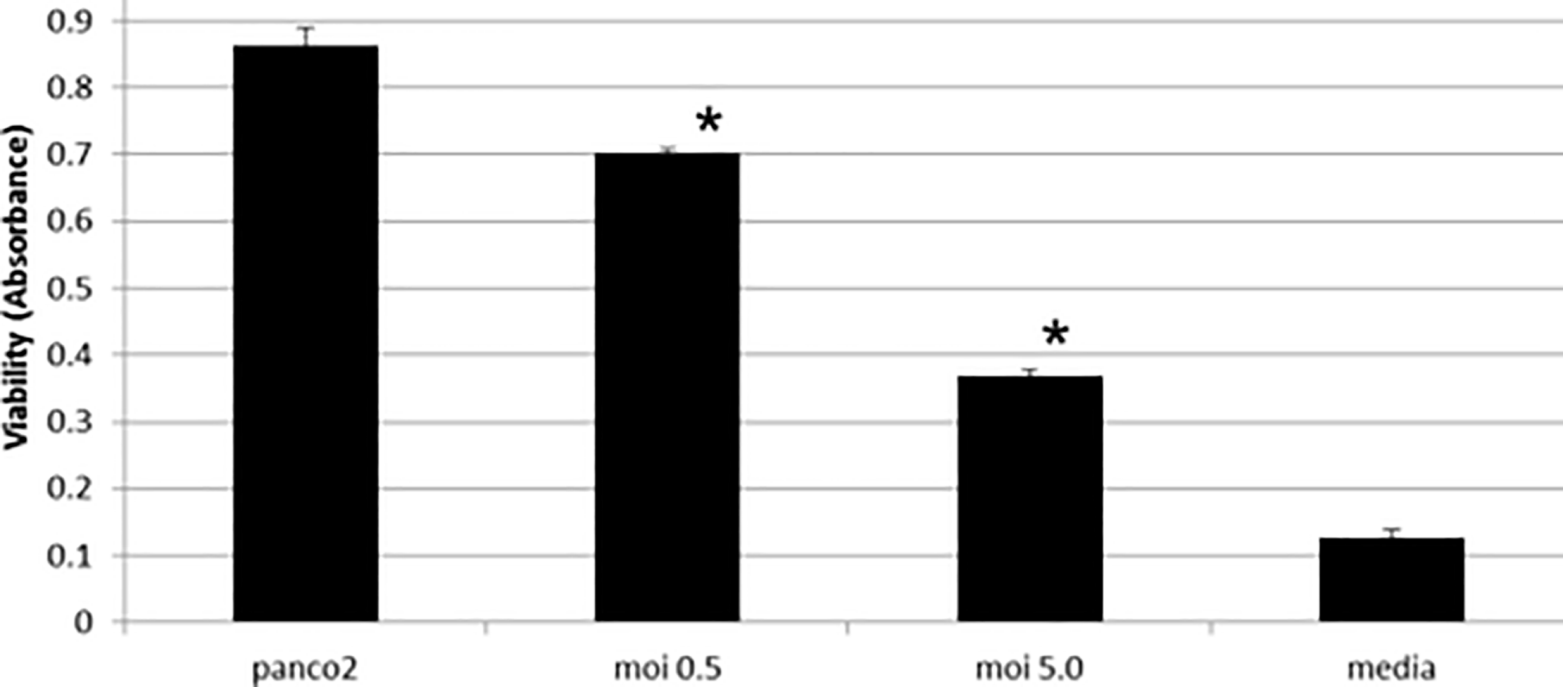
MVA kills Panc02 cells. Panc02 cells (10,000 cells/well) were plated in triplicate in a 96-well plate. MVA was added to the wells at an MOI of 0.5 or 5.0 pfu/cell. After 24 h, 10 ul MTS/PMS was added to each well, and the absorbance was read at 492 nm. Averages + SD error bars are shown. * p< 0.005. Media with no cells was used to define the background control level.

Since MVA was able to replicate in Panc02 cells, we wanted to assess whether MVA was able to kill Panc02 cells *in vitro*. Panc02 cells were uninfected or infected with an MOI of 0.5 or 5 pfu/cell and compared to media with no cells. The infection was allowed to proceed for 24 h, and MTS reagent was added to measure cell metabolism as a measure of viability. Fig 6 shows that increasing amounts of virus decreased viability of Panc02 cells. Similar results were found when infecting Panc02 cells with MVAmeso and MVAmesoA35Del viruses. These results indicate that MVA can infect Panc02 cells, replicate in them, and kill them.

### Tumor treatment

**Fig 7:**
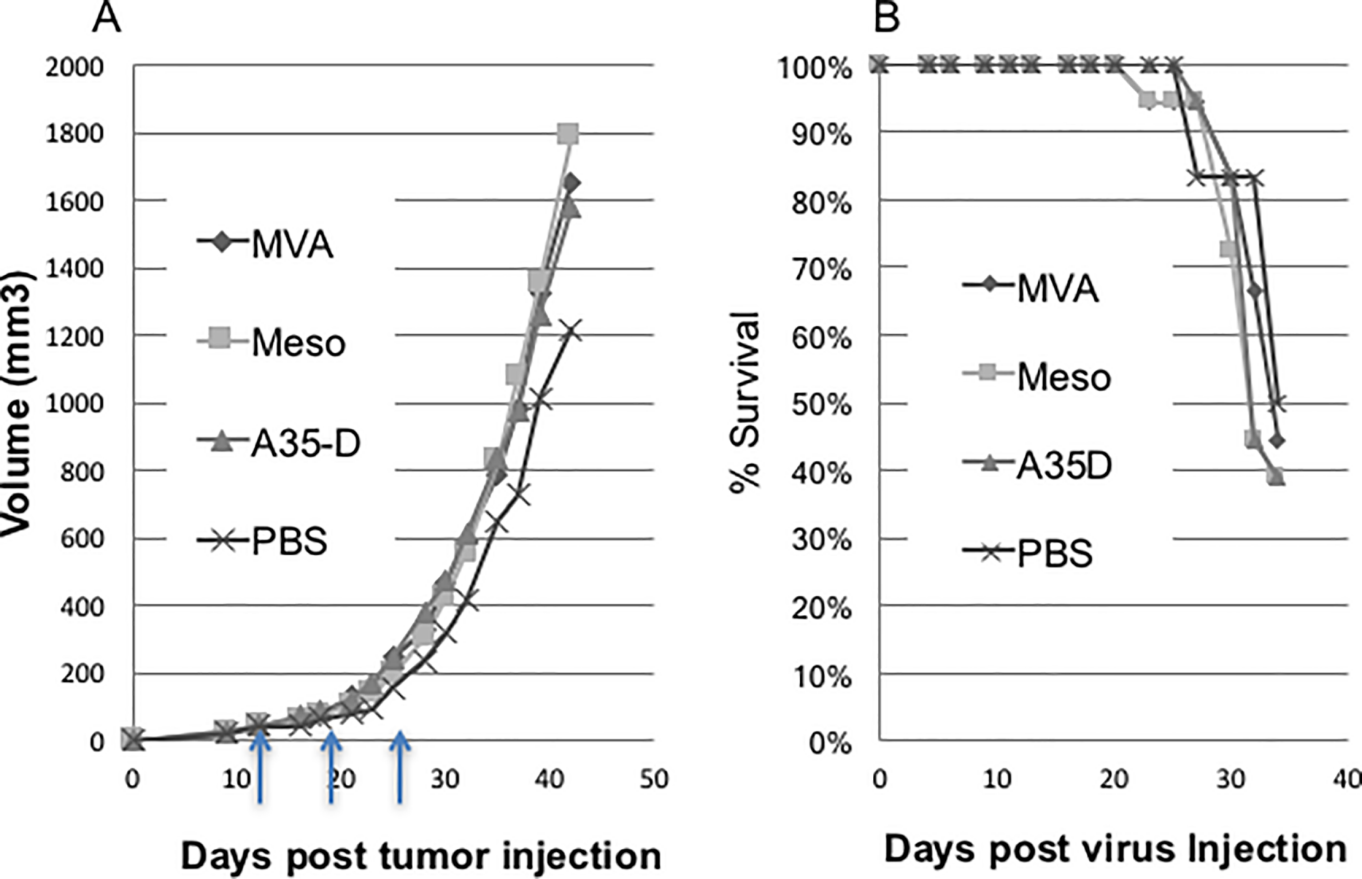
Treatment of tumor bearing mice. C57BL/6 mice were injected s.c. in one flank with 640,000 wild type Panc02 cells. At 11, 18, and 25 days (arrows) post tumor injection, mice were injected i.m. contralaterally with PBS or 7.6 x 10^7^ pfu/mouse of MVA, MVAmeso (Meso) or MVAmesoA35Del (A35D) viruses in 25 ul. (A) Tumor volumes were measured 3 times per week and survival (B) was monitored.

To test the hypothesis that administration of mesothelin expressing viruses could induce an immune response and reduce mesothelin expressing tumor growth, we established tumors in mice [7] and then treated with the MVA mesothelin viruses. To test if the removal of the immunosuppressive A35 gene improved this therapeutic vaccine efficacy, we assessed the MVAmeso virus compared to the MVAmesoA35Del virus. Tumors were established by s.c. injection of tumors in one flank of the mice. At 11, 18, and 25 days post tumor injection, mice were injected i.m. contralaterally with 25 ul of PBS, MVA, MVAmeso or MVAmesoA35Del viruses. Fig 7 shows that tumors grew similarly regardless of virus treatments (A), and survival (B) was not improved. We have previously described the construction of stable mesothelin over expressing Panc02 cells and shown that tumors formed by these cells were significantly smaller than the tumors expressing wild type levels of mesothelin [7]. We also tested whether the mesothelin expressing viruses might have greater efficacy against Panc02 cells that expressed higher levels of the Panc02 target. However, there was also no efficacy against the Panc02 tumor cells expressing increased levels of Panc02 protein, and the tumors continued to grow rapidly.

### Pre-vaccination and intratumoral injection

**Fig 8:**
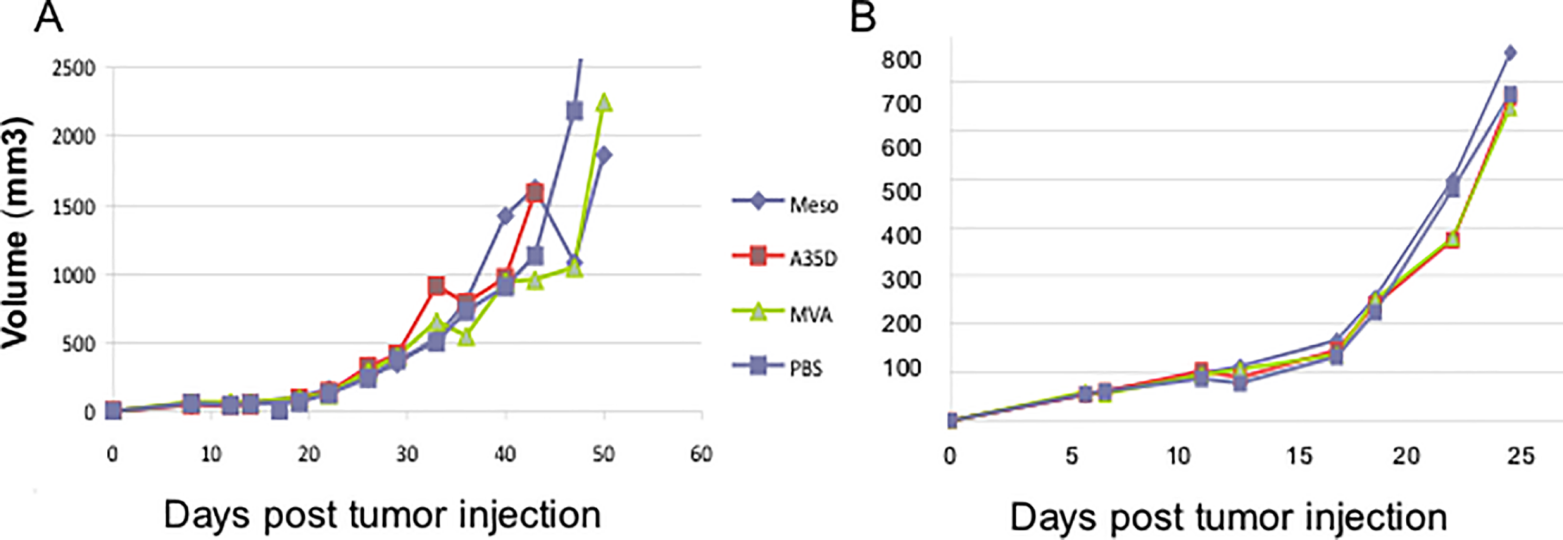
Pretreatment and intratumoral injections. (A) Mice were vaccinated 2 times and then injected with tumor cells 2 weeks later. (B) Tumors were allowed to grow to 100 mm^3^, and 3.5 x 10^6^ pfu/mouse in 25 ul of MVA, MVAmeso (meso) or MVAmesoA35Del (A35D) viruses were injected directly into tumors 3 times.

Since the Panc02 tumors grow rapidly, we tested whether inducing an immune response to mesothelin prior to tumor induction would be protective against tumor growth. Mice were injected i.m. with PBS, wild type MVA, MVAmeso, or MVAmesoA35Del, and boosted 2 weeks later. Two weeks after the boost, tumor cells were injected s.c. contralaterally in the flank. As shown in Fig 8A, the prior vaccination with MVAmeso or MVAmesoA35Del did not significantly decrease tumor growth or increase survival times. Next, we attempted direct intratumoral injections since MVA is able to replicate in and kill Panc02 cells *in vitro*. We injected tumor cells s.c. and when tumors reached 100 mm^3^, we injected PBS, wild type MVA, MVAmeso, or MVAmesoA35Del directly into tumors 3 times, 5 days apart, however, we were unable to detect a benefit (Fig 8B). We also attempted to treat stable mesothelin overexpressing Panc02 [7] tumors by intratumoral injection, however there was no apparent effect, as tumors continued to grow and mice had to be euthanized.

## Discussion

We have shown that murine mesothelin can be expressed from the MVA virus genome and detected as a ~50 kDa monomer protein on SDS-PAGE immunoblot, and a ~100 kDa presumed dimer. Mesothelin is released from cells and can be detected in the supernatants of cultured cells [7] and in much higher quantities in cells infected with MVA viruses that expresses mesothelin under a viral promoter. Injection of the mesothelin expressing viruses into animals caused no apparent ill effect to at least 4 months after vaccination, suggesting that MVAmeso vaccine is safe and does not initiate a deleterious autoimmune response. The MVAmeso vaccine induced a strong response to the mesothelin expressing Panc02 tumor cell line, only slightly smaller than the amount of anti-vaccinia virus T lymphocytes induced by injection of vaccinia virus [29]. These results indicate that the mesothelin protein was expressed in an immunogenic form by the virus in the infected mice. Furthermore, MVA virus (with and without mesothelin expression) was able to replicate in and kill Panc02 tumor cells grown *in vitro*, suggesting that it would be an effective oncolytic virus *in vivo*. Our previous results suggested that mesothelin might be a good target for control by an immune response [7], as mesothelin overexpression reliably caused a *decrease* in a heterotopic tumor growth in an immunocompetent syngeneic mouse model while *in vitro* proliferation was not inhibited [7]. Together, these results suggested that MVAmeso might be an effective treatment for Pancc02 tumors, and that the MVAmesoA35Del might be more effective given its improved immunogenic properties [31].

However, we were unable to demonstrate any efficacy of the mesothelin MVA vaccines prophylactically or therapeutically, even with multiple boosts or when injected directly into the tumors. One possibility is that the Panc02 tumor cell line is simply too aggressive for control in an animal model where the tumor microenvironment may act to protect and promote tumor cell growth [7]. While we had hoped that intratumoral injection would allow the virus to replicate in, spread, and kill tumor cells, we did not see a potent oncolytic effect in animals. Perhaps while the MVA virus can kill Panc02 tumor cells *in vitro*, the anti-viral immune response *in vivo* limits the replication and spread of the virus. An ineffective anti-tumor immune response may also be caused by the shedding of mesothelin by the tumor *in vivo* (decoy mesothelin) blocking the immune effector antibodies and T cells. Mesothelin is known to be detectable in supernatants of Panc02 cells [7] and in sera from patients with mesothelin expressing tumors [6, 48, 49]. This shed soluble mesothelin may be taken up by other cells and bind anti-mesothelin antibodies rendering them ineffective. These problems may be worsened by administration of these mesothelin-expressing viruses because they also release soluble mesothelin at high levels. Another possibility is that the shed mesothelin induces a state of anergy/tolerance in the animal, but our data show that mesothelin specific T lymphocytes are generated in these animals even though there is soluble circulating mesothelin shed from the mesothelin virus infected cells. Perhaps new developments with CART cells [10] may be able to more effectively facilitate T cell targeting and killing of cancerous cells.

Our data showed that both MVAmeso and MVAmesoA35Del vaccines were able to generate a T lymphocyte response that was activated by the Panc02 cells, and the MVAmesoA35Del induced a stronger response, however it may be possible that the Panc02 cells have developed mutations that protect them from effective cytotoxic T lymphocyte killing, even if T lymphocytes are appropriately expanded and targeted to recognize them. Tumors that develop in humans and animals have already been highly selected *in vivo* to avoid the immune responses that control cancers. Further improvements in these oncolytic therapeutic vaccines may be made by including cytokines such as IL-10 and GMCSF, which have been reported to improve oncolytic potential of vaccinia virus and ultimately host survival [50, 51]. In addition, depletion of T regulatory cells may counteract homeostatic or cancer cell driven immunosuppression and enhance the development of mesothelin-specific CD8 T cells capable of killing tumor cells [52, 53]. Although some results suggest that immune responses can be counter-productive: the anti mesothelin immune response may induce autoantibodies to the target antigen as well as other proteins by epitope spreading, and these antigen-spreading autoantibodies may cause increased damage to the animal. It was previously shown that antigen spreading was associated with a trend toward decreased survival in patients with prostate cancer treated with a recombinant viral vaccine [46].

Finally, given the difficulties with targeting an antigen like mesothelin that is shed, it may be more effective to target an antigen that is not shed. Recent results targeting a different pancreatic tumor associated antigen, the oncofetal fucose-rich glycovariants of the pathological bile salt-dependent lipase, suggest it may be a superior tumor target antigen [54].
